# Proton Nuclear Magnetic Resonance-Spectroscopic Discrimination of Wines Reflects Genetic Homology of Several Different Grape (*V*. *vinifera *L.) Cultivars

**DOI:** 10.1371/journal.pone.0142840

**Published:** 2015-12-11

**Authors:** Boran Hu, Yaqing Yue, Yong Zhu, Wen Wen, Fengmin Zhang, Jim W. Hardie

**Affiliations:** 1 College of Food Science and Engineering, Yangzhou University, Yangzhou, Jiangsu, China; 2 College of Tourism and Gastronomy, Yangzhou University, Yangzhou, Jiangsu, China; 3 Testing Center of Yangzhou University, Yangzhou, Jiangsu, China; 4 Northern Melbourne Institute of TAFE, Epping, Victoria, Australia; Imperial College London, UNITED KINGDOM

## Abstract

**Background and Aims:**

Proton nuclear magnetic resonance spectroscopy coupled multivariate analysis (^1^H NMR-PCA/PLS-DA) is an important tool for the discrimination of wine products. Although ^1^H NMR has been shown to discriminate wines of different cultivars, a grape genetic component of the discrimination has been inferred only from discrimination of cultivars of undefined genetic homology and in the presence of many confounding environmental factors. We aimed to confirm the influence of grape genotypes in the absence of those factors.

**Methods and Results:**

We applied ^1^H NMR-PCA/PLS-DA and hierarchical cluster analysis (HCA) to wines from five, variously genetically-related grapevine (*V*. *vinifera*) cultivars; all grown similarly on the same site and vinified similarly. We also compared the semi-quantitative profiles of the discriminant metabolites of each cultivar with previously reported chemical analyses. The cultivars were clearly distinguishable and there was a general correlation between their grouping and their genetic homology as revealed by recent genomic studies. Between cultivars, the relative amounts of several of the cultivar-related discriminant metabolites conformed closely with reported chemical analyses.

**Conclusions:**

Differences in grape-derived metabolites associated with genetic differences alone are a major source of ^1^H NMR-based discrimination of wines and ^1^H NMR has the capacity to discriminate between very closely related cultivars.

**Significance of the Study:**

The study confirms that genetic variation among grape cultivars alone can account for the discrimination of wine by ^1^H NMR-PCA/PLS and indicates that ^1^H NMR spectra of wine of single grape cultivars may in future be used in tandem with hierarchical cluster analysis to elucidate genetic lineages and metabolomic relations of grapevine cultivars. In the absence of genetic information, for example, where predecessor varieties are no longer extant, this may be a particularly useful approach.

## Introduction

Grape wine is a complex mixture of several hundred components present in various concentrations. It contains many metabolites from grapes but most chemicals in wine are products of alcoholic and malolactic fermentations. The main compounds are water, ethanol, glycerol, sugar and organic acids. Other compounds, such as phenols, amino acids and so-called secondary metabolites, are present in much lower concentrations. Many factors, including microbial ecology of the ferment, geographical origin, grape cultivar and winemaking technologies contribute to the composition and content of metabolites in wines and affect the quality of wines [1].

Due to the increasing interest in the qualities and provenance of grape cultivars and wines by consumers, regulatory authorities and producers, it is of commercial importance to develop fast and accurate methods to distinguish wines according to grape cultivar, geographic origin and other product-defining features.

NMR has been used increasingly in the characterization and quality control of wine and food [2,3]. Although the sensitivity of NMR is dependant on magnetic strength of the instrument and some NMR experiments do not yield more or better information than alternative analytical techniques, NMR still has many advantages over other methods. NMR allows rapid, simultaneous, measurement of a large number of organic compounds in complex mixtures and requires minimal pre-treatment of samples; thus reducing errors introduced by pre-treatment and preservation of the samples ([4–6]). Multivariate data analyses including principal component analysis (PCA) and partial least squares discriminant analysis (PLS-DA) are used to decrease the dimensionality of multivariate data and classify items according to inherent patterns in the data. Hierarchical cluster analysis (HCA) allows segregation of groups of items according to the degree of similarity between them.

Proton nuclear magnetic resonance (^1^H NMR) spectroscopy coupled with PCA or PLS-DA has been used to discriminate between wines produced from the same cultivar grown in different geographic regions [7–9], two or more cultivars grown in the same geographic region [10,11]—including under different seasonal (‘vintage’) conditions [12,13], several cultivars of different *Vitis* species grown in different geographic regions [14] and binary blends of single cultivar wines [15]. Notably, Pereira et al. found that within a season, across three contrasting soil types within a vineyard, genotypic discrimination of cultivars by ^1^H HMR spectra was dominated by variation in soil type and that, between seasons, variability induced by the climate was most dominant.

Recent genomic studies (Robinson et al. [16] and references cited therein) have revealed for the first time the genetic relations of some commercially important cultivars. This allowed us to determine the extent to which ^1^H NMR spectra, in the absence of confounding environmental and vinicultural factors, reflect inherent genotypic differences between cultivars. He found that three of the cultivars, Cabernet Sauvignon, Merlot and Ruby Cabernet, are closely related genetically by sharing Cabernet Franc either as a parent (Cabernet Sauvignon and Merlot) or a grandparent (Ruby Cabernet). The other two cultivars, Zinfandel (syn. Tribidrag, Crljenak Kastelanski, Primativo) and Syrah (syn. Shiraz), are more distantly related, both to each other and the ‘Cabernet Franc’ group [16]. But wines of Ruby Cabernet and Zinfandel have not been included in previously reported NMR studies. Accordingly we applied ^1^H NMR with pattern recognition analyses to discriminate wines vinified similarly from five different grape cultivars of the same species, *Vitis vinifera* L., grown similarly on the same soil type in the same vineyard and harvested at similar ripeness. To corroborate our evidence of genetic discrimination, we compared the cultivar-related profiles in the levels of discriminant grape metabolites with reported chemical analyses of those cultivars.

We show the capacity of ^1^H NMR, coupled with PCA, PLS-DA and HCA. To discriminate between wines on the basis of the genetic relatedness of the cultivars, we also report the discriminant compounds and their general relative, cultivar-based profiles.

## Materials and Methods

We state clearly that no specific permissions were required for these activities, because the brewing company issued the permission. We confirm that the field studies did not involve endangered or protected species.

All wine samples are produced in Shacheng Manor Wine Co. Ltd., Hebei province, China. After fermentation, we got 3 parallel samples of each wine from the sampling mouth of a 30 ton fermenting tank, each replicate sample was funnelled, using a 750 mL funnel, into a brown glass bottle which was then sealed with a cork and transported to the laboratory, and then stored at -4°C.

### Sample origin and chemical analysis

The wine samples were of Cabernet Sauvignon, Merlot, Ruby Cabernet, Syrah and Zinfandel, each of the 2010 vintage and grown, non-grafted, in a single vineyard of uniform soil type in the Shacheng region of Hebei Province, China. The grapes of each cultivar were harvested at similar concentrations of reducing sugar (Table 1). Four replicate wines of each cultivar were vinified with the same yeast (Lalvin CY 3079), without chemical adjustment other than addition of potassium metabisulfite (50 mg/L) and they were not matured in contact with wood. The physical and chemical features of the wines, analyzed according to the Chinese national standard (GB/T 15038–2006), are presented in Table 2.

**Table 1:** Grape cultivars and fruit composition at harvest.

Cultivar	Harvest Date	Reducing Sugar (g/L)	Titratable Acidity (g/L)	pH
Cabernet Sauvignon	Oct. 6	226.2	6.8	3.3
Merlot	Sept. 25	215.6	6.4	3.4
Ruby Cabernet	Oct. 3	211.5	6.2	3.4
Syrah	Oct. 4	225.1	6.7	3.3
Zinfandel	Sept. 21	213.4	6.3	3.4

**Table 2:** Physical and chemical features of the wines *.

	Cultivars				
	Cabernet Sauvignon	Ruby Cabernet	Zinfandel	Merlot	Syrah
Alcohol content %vol	12.8	12.1	11.8	12.4	12.8
Residual sugar(glucose) g/L	2.11	2.07	2.03	2.11	2.23
Total acid g/L	5.7	5.8	5.5	6	6.1
Volatile acid g/L	0.48	0.45	0.51	0.48	0.48
Dry extract g/L	24.8	25.8	23.8	24.9	25.8
pH	3.55	3.56	3.54	3.51	3.46
Total SO_2_ mg/L	72	82	81	79	76
Free SO_2_ mg/L	31	28	34	30	34
Methanol mg/L	221	191	201	214	199
Fe^3+^ mg/L	1.2	2.1	1.8	2.2	1.8
Cu^2+^ mg/L	0.05	0.05	0.08	0.06	0.07
K^+^ mg/L	882	931	999	988	972
Ca^2+^ mg/L	85	86	97	95	85
Tartaric acid g/L	2.42	2.37	2.28	2.24	2.41
Citric acid g/l	0.33	0.28	0.27	0.35	0.2
Lactic acid g/L	2.09	2.98	2.64	2.78	2.14
Colour tone	11.7	11.8	10.8	12.7	12.9
Colour tint	0.78	0.82	0.82	0.81	0.78

*Methods of determination of physical and chemical features accorded with China National Standard GB/T 15038–2006

### NMR sample preparation

10mL of wine of each replicate was centrifuged at 4,000rpm for 10min. Supernatants (3 mL) were frozen at -70°C for 10h and then lyophilized for 48h. The lyophilized product was dissolved in 99.9% deuterium oxide (630 μL, D_2_O) and 0.75% 4, 4-dimethy-l-4-silapentane-1-sulphonic acid (70 μL, DSS) and centrifuged at 13,000rpm for 10min. The supernatant (500 μL) was placed in a 5mm NMR tube. DSS provided a chemical shift reference (δ = 0) and the internal standard for quantitative analysis. D_2_O provided a field frequency lock signal.

To confirm the influence of pre - ^1^H NMR sample lyophilization the following procedure was applied: 3mL of wine (Cabernet Sauvignon) was centrifuged at 12,000rpm for 20min. The supernatant (450 μL) was mixed with 50 μL of a solution of 0.75% 4, 4-dimethy-l-4-silapentane-1-sulfonic acid (DSS) in 99.9% deuterium oxide (D_2_O) in a 5mm NMR tube. Four replicate samples of wine treated this way were subsequently spectrally compared with lyophilized samples after suppression of the water peaks.

### NMR spectroscopy

^1^H NMR spectra were recorded on a Bruker AVANCE 600 spectrometer, operating at 600.13MHz 1H frequency and a temperature of 298K, using a ^1^H {13C/15N} probe. A NOESYPRESAT pulse sequence was used to suppress the residual water signal. A total of 256 transients were collected into 32,000 complex data points with a spectral width of 7183.9Hz, with a relaxation delay of 2s, an acquisition time of 2.3s, and a mixing time of 100ms. The NMR spectra were processed with a line-broadening factor of 0.3Hz prior to Fourier transformation. ^1^H-^1^H correlation spectroscopy (COSY) data were acquired with a time domain data matrix of 2048 by 512 and a mixing time of 80ms.

### NMR data reduction

All of the NMR spectra were phase and baseline corrected by AMIX and then the NMR spectral data were reduced into 0.005ppm spectral buckets. The regions corresponding to water (4.6–4.8ppm), incompletely removed ethanol (1.18–1.22 ppm and 3.57–3.72 ppm) and DSS (-0.5–0.5 ppm, 1.74–1.84 ppm and 2.90–2.95 ppm) were removed. The datasets were then imported into SIMCA-P version 12.0 for multivariate statistical analysis.

### Multivariate data analysis

Principal component analysis (PCA), an unsupervised pattern recognition method, i.e. without preliminary hypotheses, was used to examine the intrinsic variation in the dataset. To maximize the separation between samples partial least squares discriminant analysis (PLS-DA) was applied. PLS-DA can be regarded as a supervised derivative of PCA that provides the maximum covariance between measured data (X variable, metabolites in NMR spectra) and the response variable (Y variable, NMR spectral intensities), it can selectively extract the Y-relevant variation of variables, but also ensure the maximum correlation between them. The Hotelling’s T^2^ region, shown as an ellipse in score plots of the models, defines the 95% confidence interval of the modeled variation [17]. After orthogonal signal correction (r = 4) was applied to eliminate information that did not contribute to the discrimination, PLS-DA score plots from the ^1^H NMR spectra of wines of the five cultivars were generated in pairwise comparisons. The quality of the models is described by the R^2^X and Q^2^ values. R^2^X is defined as the proportion of variance in the data explained by the models and indicates goodness of fit. Q^2^ is defined as the proportion of variance in the data predictable by the model and indicates predictability.

### Hierarchical cluster analysis

^1^H NMR data for four replicate wine samples of each cultivar was imported into IBM SPSS 19.0 for hierarchical cluster analysis to characterize the degree of metabolic similarity between the cultivars.

### Assignment of metabolites

**Fig 1:**
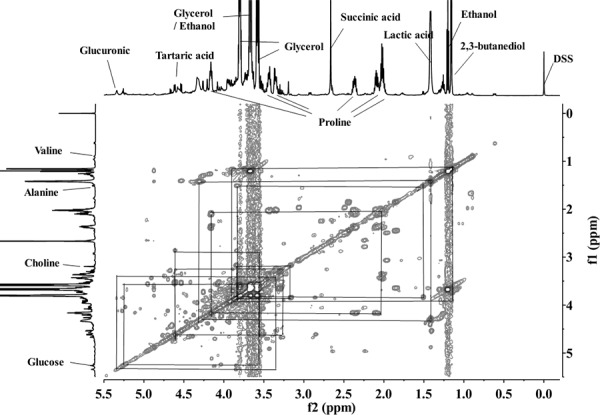
The ^1^H-^1^H COSY NMR spectrum of lyophilized Cabernet Sauvignon dry red wine.

From the ^1^H NMR and ^1^H-^1^H COSY spectra, e.g. Fig 1, and according to chemical shifts reported by Larsen et al. [18]; Lee et al. [19]; Pereira et al. [20]; and Son et al. [7], we identified and assigned the major resonances for 17 components in the wines (Table 3). Identification of corresponding loading plot signals indicated the PLS-DA discriminant metabolites.

**Table 3:** Metabolites and their ^1^H chemical shifts identified by 600 MHz ^1^H NMR^*^.

No.	Compound	Molecular formula	^1^H NMR chemical shift
1	Ethanol	C_2_H_5_OH	1.19(t,C2H_3_),3.66(q,C1H_2_)
2	Methanol	CH_3_OH	3.36(s,CH_3_)
3	2,3—butanediol	C_4_H_10_O_2_	1.15(d,C1H_3_),3.88(q,C4H_3_)
4	Glycerol	C_3_H_8_O_3_	3.56(q,C2H_2_),3.65(q,C3H_2_),3.78(m,C1H)
5	Acetic acid	CH_3_COOH	2.07(s,C3H_3_)
6	Lactic acid	C_3_H_6_O_3_	1.38(d,C3H_3_),4.29(m,C2H)
7	Tartaric acid	C_4_H_6_O_6_	4.53(s,C2H+C3H)
8	Succinic acid	C_4_H_6_O_4_	2.67(s,C2H_2_+C3H_2_)
9	*β*-glucose	C_6_H_12_O_6_	4.64(d,*α-*C1H,ring),3.24(dd,u,C2H,ring)3.54(dd,u,C3H,ring)
10	*α*-glucose	C_6_H_12_O_6_	5.26(d,*β-*C1H,ring),3.55(dd,u,C2H,ring)
11	*α*-D-glucuronic	C_6_H_10_O_7_	5.34(d,C1H,ring)
12	*β*-D-glucuronic	C_6_H_10_O_7_	4.58(d,C1H,ring)
13	Valine	C_5_H_11_NO_2_	0.89(d,C4H_3_),0.95(d,C5H_3_)
14	Alanine	C_3_H_7_NO_2_	1.52(d, *β-*C3H_3_),3.88(q, u, *α-*CH)
15	Proline	C_5_H_9_NO_2_	2.02(m,u,*γ*-CH_2_),2.07(m,u,*β*-CH),2.36(m,u,*β'*-CH),3.36(m,u,*δ*-CH),3.42(m,u,*δ*-CH),4.17(m,u,*α*-CH)
16	Choline	C_5_H_15_NO_2_	3.19(s,N-CH_3_),3.49(t,u, *α*-CH_2_),3.83(t, *β*-CH_2_)
17	Gallic acid	C_7_H_6_O_5_	7.16(s,C2H+C6H)

*Letters in parentheses indicate the peak multiplicities

s, singlet

d, doublet

t, triplet

dd, doublet of doublet

tt, triplet of triplets

q, quartet; and

m, multiplet.

### Relative amounts of discriminant metabolites between cultivars

To provide an indication of the relative amounts of each of the discriminant metabolites between the cultivars and to allow some general hierarchical trends in those metabolites according to cultivar, a matrix of cultivar-discriminating compounds was constructed using the relative peak heights of compounds represented in the pairwise comparison loading plots. Ranking within each hierarchy was established on a semi-quantitative basis with regard only to the comparative magnitude (i.e. greater or lesser) of the peak heights in each possible pairwise comparison.

## Results

### Comparison of wine samples with and without lyophilization

**Fig 2:**
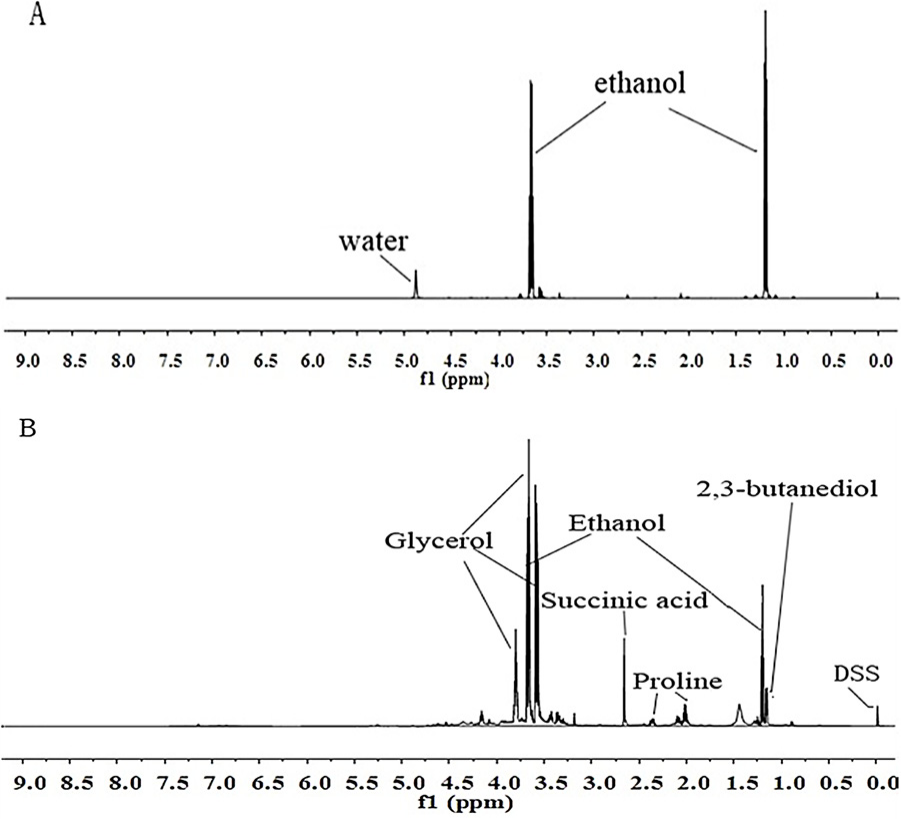
^1^H NMR spectrum of Cabernet Sauvignon wine with NOESYPRESAT water peak suppression, (A) sample not lyophilized, (B) sample lyophilized.

Fig 2 presents the comparison of NOESYPRESAT water peak—suppressed ^1^H NMR spectra of Cabernet Sauvignon wine with and without lyophilization to remove water. In the spectra of samples without lyophilization the signals of ethanol and glycerol were so intense that they obscured the signal of other less abundant components. That made the identification and assignment of minor components difficult. In the spectra of lyophilized samples, even though a small amount of water and ethanol remained, the minor components were not obscured and were more readily identified.

### Multivariate analysis

**Fig 3:**
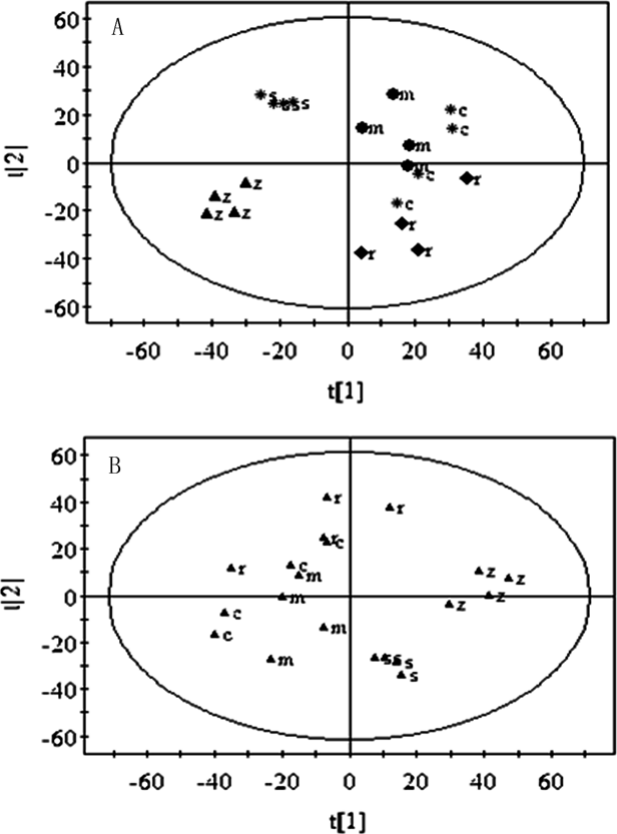
Comparison of wine discrimination derived from the ^1^H NMR spectra. (A) PCA Scores Plot (t1/t2), R^2^X = 0.959, Q^2^ = 0.872. PC1/PC2 accounted for 65.9% of the total variance. (B) PLS-DA Scores Plot (t1/t2), R^2^X = 0.959, R^2^Y = 0.991, Q^2^ = 0.954. PC1/PC2 accounted for 65.5% of the total variance. Grape cultivars: m, Merlot; s, Syrah; z, Zinfandel; r, Ruby Cabernet; c, Cabernet Sauvignon.

All of the NMR spectra were pre-treated by AMIX and then imported into SIMCA-P version 12.0 for PCA and PLS-DA. PCA of ^1^H NMR spectra revealed a general clustering of the replicate wines by cultivar. The score plot of the first two principal components (PCs) is shown in Fig 3A. The first two PCs accounted for 65.9% of the total variance. On this analysis wines of both Zinfandel and Syrah were characterized by positive score clusters on PC1 in contrast to the other cultivars that were generally characterized by negative scores on that PC. Wines of the other cultivars were less strongly discriminated but on PC2 the scores for Ruby Cabernet were positive while those for Merlot were mostly negative and those of Cabernet Sauvignon were both positive and negative.

PLS-DA of ^1^H NMR spectra (Fig 3B) provided similar but slightly greater discrimination than PCA, PC1/PC2 mapping accounted for 65.5% of total variability. With the exception of Cabernet Sauvignon wines, the scores of each cultivar cluster were more exclusively positive or negative on PC2 than in the PCA analysis.

### Hierarchical cluster analysis

**Fig 4:**
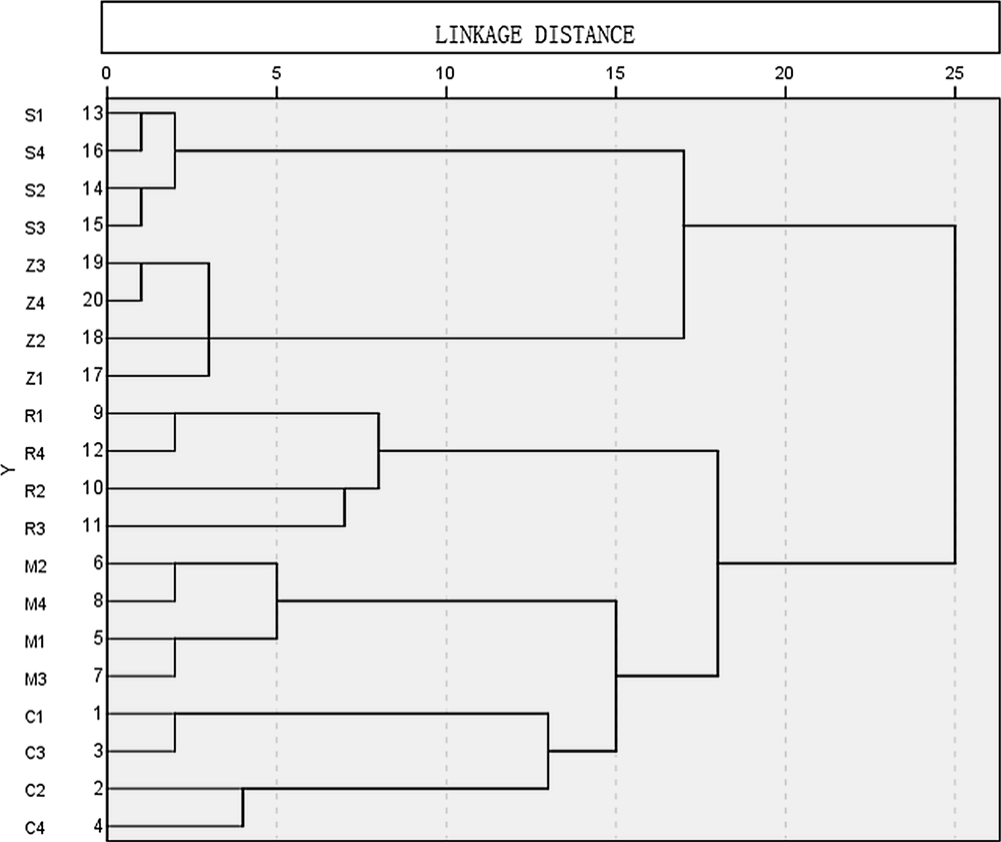
Dendrogram of wines of five cultivars (M, Merlot; C, Cabernet Sauvignon; R, Ruby Cabernet; Z, Zinfandel; S, Syrah), (4 replicates of each), based on multidimensional analysis of metabolites detected by ^1^H NMR spectroscopy.

Following aggregation of the replicates of each cultivar, the successive nodes of the dendrogram aggregated Merlot and Cabernet Sauvignon, the ‘Cabernet Franc’ group, and Syrah and Zinfandel respectively (Fig 4). There was clear separation between each of the groups.

### Pairwise comparison of wines and discriminating metabolites

**Fig 5:**
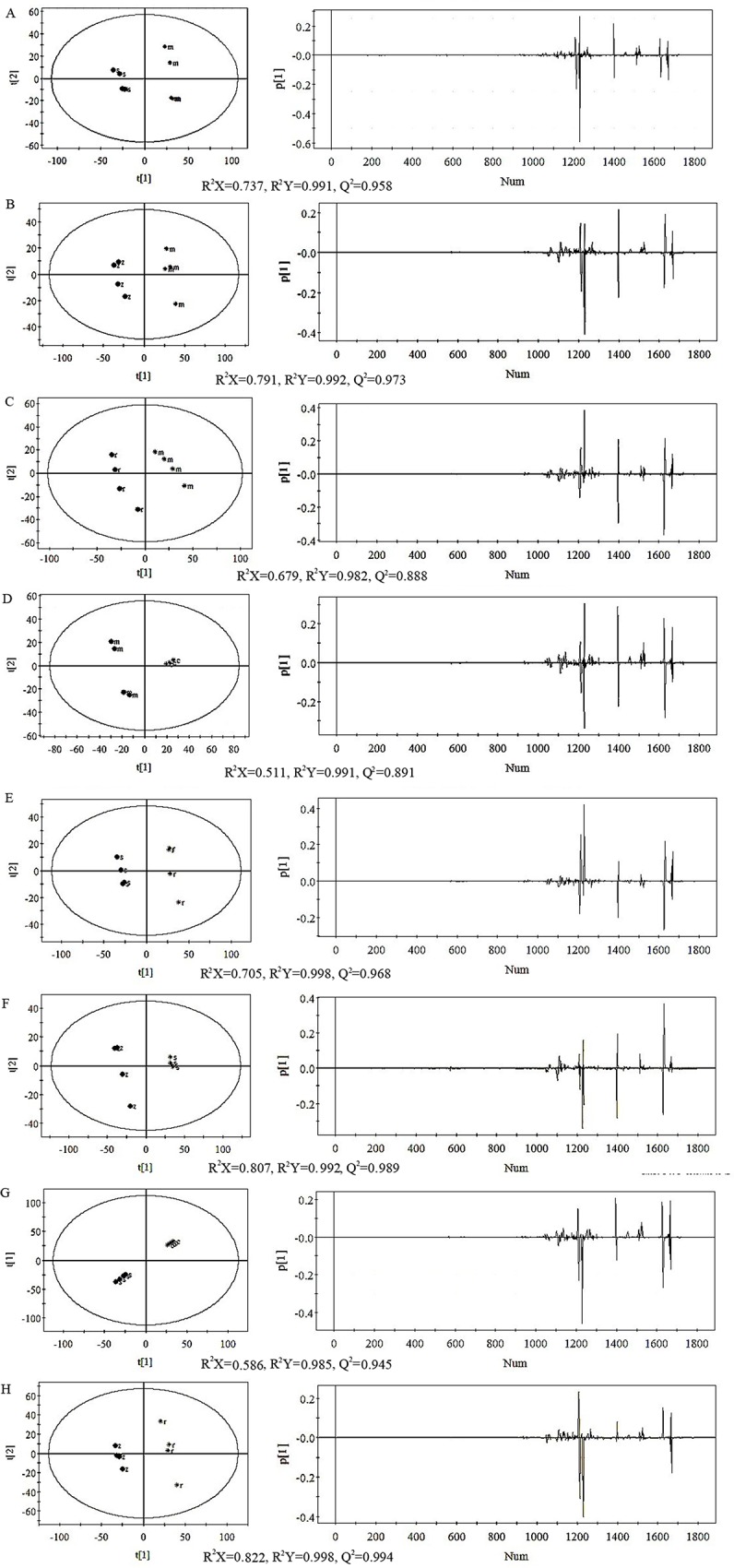
PLS-DA score plots, loading plots and correlation parameters derived from the ^1^H NMR spectra of wines as pair wise comparisons (A) Merlot (m) and Syrah (s), R^2^X = 0.737, R^2^Y = 0.991, Q^2^ = 0.958. PC1/PC2 variance accounted for 72.1%. (B) Merlot (m) and Zinfandel (z), R^2^X = 0.791, R^2^Y = 0.992, Q^2^ = 0.973. PC1/PC2 accounted for 79.1%. (C) Merlot (m) and Ruby Cabernet (r), R^2^X = 0.679, R^2^Y = 0.982, Q^2^ = 0.888. PC1/PC2 variance accounted for 67.9%. (D) Merlot (m) and Cabernet Sauvignon (c), R^2^X = 0.511, R^2^Y = 0.991, Q^2^ = 0.891. PC1/PC2 variance accounted for 51.4% (E) Syrah (s) and Ruby Cabernet (r), R^2^X = 0.705, R^2^Y = 0.998, Q^2^ = 0.968. PC1/PC2 variance accounted for 70.5%. (F) Syrah (s) and Zinfandel (z), R^2^X = 0.807, R^2^Y = 0.992, Q^2^ = 0.989. PC1/PC2 accounted for; 80.7%. (G) Syrah (s) and Cabernet Sauvignon (c), R^2^X = 0.586, R^2^Y = 0.985, Q^2^ = 0.945. PC1/PC2 variance accounted for 56.7%. (H) Ruby Cabernet (r) and Zinfandel (z), R^2^X = 0.822, R^2^Y = 0.998, Q^2^ = 0.994. PC1/PC2 variance accounted for 80.2%. (I) Cabernet Sauvignon (c) and Zinfandel (z), R^2^X = 0.897, R^2^Y = 0.994, Q^2^ = 0.983. PC1/PC2 variance accounted for 89.7%. (J) Cabernet Sauvignon (c) and Ruby Cabernet (r), R^2^X = 0.775, R^2^Y = 0.995, Q^2^ = 0.976. PC1/PC2 variance accounted for 74.8%.

Pairwise PLS-DA score plots derived from the ^1^H NMR spectra of wines of the five cultivars showed clear discrimination (i.e. high values of the regression coefficients R^2^X, R^2^Y and cumulative Q^2^; the proportion of the variation of Y predicted by the model) between wines of each cultivar by the first component, and the loading plots showed the metabolites that contributed to the discrimination (Fig 5). In the loading plot each peak represents an integral region of the NMR spectra and the height of the peak corresponds to a contribution coefficient of the region to the separation shown in the score plot. The discriminating compounds were grape metabolites, viz. proline, valine, total phenols (mostly Gallic acid), tartaric acid and glucose; and vinification products, viz. glycerol, 2, 3—butanediol, succinic acid, lactic acid, acetic acid and valine.

Table 4 presents the matrix of cultivar-discriminating compounds derived from the pairwise comparison of peak heights from the loading plots. The matrix provides an indication of the relative levels of many of those compounds and allows some general hierarchical classifications by cultivar. Some compounds were insufficiently represented within the matrix to allow hierarchical classification.

**Table 4:** Matrix of cultivar-discriminating compounds^*^.

	Cabernet Sauvignon	Merlot		Ruby Cabernet		Syrah		Zinfandel	
	Compounds that quantitatively exceed those of the cultivar in left hand column								
Cabernet Sauvignon			lactic acid,glucose,valine		lactic acid,tartaric acid,glucose,valin,phenols		2,3-butanediol,glycerol,lactic acid,tartaric acid,glucose		glycero,tartaric acid,valine,gallic acid
Merlot	2,3-butanedio,glycero,tartaric acid,succinic acid,proline				lactic acid,succinic acid,phenols		ethanol,2,3-butanediol,glycerol		2,3-butanediol,glycerol,succinic acid,glucose
Ruby Cabernet	2,3-butanediol,acetic acid,succinic acid,glucose,proline		2,3-butanediol,glycerol,acetic acid,tartaric acid,proline				2,3-butanediol,glycerol,tartaric acid		2,3-butanediol,glycero
Syrah	acetic acid,succinic acid,proline		tartaric acid,glucose,proline,phenols		lactic acid,succinic acid,proline,phenols				glycerol,tartaric acid,succinic acid,glucose
Zinfandel	2,3-butanediol,acetic acid,succinic acid,glucose,proline,phenols		acetic acid,tartaric acid,valine,proline,phenols		lactic acid,tartaric acid,succinic acid,glucose,valine,proline,phenols		2,3-butanediol,acetic acid		

*Compounds determined from loading variables from pair wise PLS-DA. Contents of compounds within cultivar columns exceed those of the cultivar shown in the column to the left.

## Discussion

The high resolution of ^1^H NMR spectral data coupled with either PCA or PLS-DA allowed discrimination between wines of Syrah and Zinfandel and between each of those two cultivars and each of the representatives of the Cabernet Franc group, viz. Cabernet Sauvignon, Merlot and Ruby Cabernet. Among those three cultivars there was clear discrimination between Merlot and Ruby Cabernet, and Cabernet Sauvignon clustered between them. While several previous NMR studies of wine ─ including wine from Merlot, Cabernet Sauvignon and Cabernet Franc grown on three different soil types within a single vineyard in Saint Emilion, southwestern France [13] ─ have shown clear discrimination between the cultivars, their genetic relations were not considered. Significantly, in the present study the degree of discrimination between all cultivars reflected a broad range of genetic homology. Ruby Cabernet, bred by H. P. Olmo in California, is well known as the progeny of Cabernet Sauvignon and the distantly related Carignan (syn. Cariñena) [21]. Recent genomic analyses have shown that Cabernet Sauvignon and Merlot have a common parent; Cabernet Franc,a cultivar grown in southwestern France from the seventeenth century [22,23]. The other two wines were from more distantly related cultivars, viz. Zinfandel, grown in Croatia as Tribidrag or Crljenak Kastelanski from the seventeenth century [16], and Syrah, a cultivar from southeastern France [24]. The discrimination of Ruby Cabernet samples from the other representatives of the ‘Cabernet Franc’ group almost certainly reflects metabolic differences arising from the intermixing of the genes of ‘Cabernet Franc’ group of south-western France and Carignan, a cultivar believed to have originated in Spain [16]. Significantly Pereira et al. (2007) found that for similarly vinified wines both seasonal climate and soil type influenced the degree of discrimination between cultivars [13]. Thus on the basis of differences in those factors we would expect differences evident in the degree of discrimination of Cabernet Sauvignon and Merlot in our study and theirs.

Hierarchical cluster analysis revealed groupings that not only reflect the genetic heritage of the cultivars but also represented the degree of similarity between them. The cultivars segregated into two groups, viz. the ‘Cabernet Franc’ group comprising Cabernet Sauvignon, Merlot and Ruby Cabernet, and the ‘others’; Zinfandel and Syrah. Notably, within the ‘Cabernet Franc’ group Merlot and Cabernet Sauvignon which have the common parent Cabernet Franc, were more closely linked to each other than to Ruby Cabernet; despite Cabernet Sauvignon being a parent of Ruby Cabernet. As we have suggested, this is possibly the result of human-mediated breeding which introduced the more distantly related cultivar, Carignan, into the group. Within the ‘other’ group it appears that, in contrast to the ‘Cabernet Franc group, Zinfandel and Syrah do not share close relatives.

Most significantly, the general correlation between degree of discrimination and genetic relatedness across a wide range of genetic homology represented by the cultivars in this study, including discrimination between very closely related cultivars, establishes that genetic variation among grape cultivars can account for the discrimination of wine by ^1^H NMR. The grape metabolites that most represented that variation were proline, valine, total phenols (mostly Gallic acid), tartaric acid and glucose (see in S1 File.). Proline, which unlike other amino acids, usually (but not always) remains unconsumed by yeast during fermentation, was the predominant amino acid found in the NMR spectrogram. It is well known that among the red wine cultivars Cabernet Sauvignon and Merlot have particularly high levels of proline [25,26]. Ough (1968) showed that the general order of abundance (% of total juice N) of proline among the ‘Cabernet Franc group’ is Cabernet Sauvignon > Merlot = Cabernet Franc > Ruby Cabernet. Similarly in our study, the content of proline was highest in wines of the ‘Cabernet Franc group’ and the hierarchical order of abundance from highest to lowest was Cabernet Sauvignon > Merlot > Ruby Cabernet > Syrah = Zinfandel. This finding accords with that of Son et al. (2008) who found that proline was an important variable in discriminating Australian Cabernet Sauvignon from Australian Shiraz wines by ^1^H NMR[7]. Although valine was a discriminant compound in our study, levels of this amino acid are determined by both grape and microbial metabolism [5] and there was insufficient evidence to identify it as a useful discriminating variable in terms of genetic relatedness of cultivars. Variation in glucose content among grape cultivars at similar total sugar content is well known but variation among yeast strains in the capacity to ferment glucose and fructose is also known [27]. Thus variation in the glucose content of wine in our study may not be attributable solely to genetic differences among the grape cultivars.

The levels of phenols (mono and polymeric) in wine are affected, not only by grape cultivar but also by region, cultural practices, fermentation processes, maturation with wood contact and wine age [1]. Polyphenolic compounds, in particular, are not easily assigned by NMR due to their complex s tructures [2]. Nevertheless total phenols (mostly Gallic acid) contributed to the discrimination of wines by cultivar in this study. The order of abundance, viz. Ruby Cabernet > Cabernet Sauvignon > Merlot > Syrah > Zinfandel, generally accord with analytical reports which indicate relative levels of total phenols in wines of those cultivars as follows: Ruby Cabernet > Cabernet Sauvignon ≥ Merlot ≥ Cabernet Franc > Syrah ≥ Zinfandel; although the position of Syrah from those reports is not completely clear [28–34].

Tartaric acid is the major grape acid in wine. The concentration of tartaric acid in grapes depends on cultivar, ripening conditions and fruit maturity. Although tartaric acid is also commonly added prior to fermentation in order to adjust the pH and acidity, the wines examined in this study were not treated this way. In our study, tartaric acid was a discriminant compound however there was insufficient evidence to establish a hierarchy among the cultivars based on tartaric acid. It is well known that the content of tartaric acid is not constant during wine fermentation because of the formation of potassium bitartrate and calcium tartrate which tend to precipitate in wines. Generally, as Son et al. [14] noted, tartaric acid is unlikely to be a useful biomarker to characterize wines of hetereogenous vinification processes but as we have shown, without addition during vinification, it may be a useful component for discrimination wines by genetic origin.

Although compounds of grape origin obviously provide the foundation for discrimination between cultivars, the PCA and PLS-DA approach relies on discrimination on the basis of maximizing differences in chemical profiles in both qualitative and quantitative terms regardless of source. The discriminating compounds arising during alcoholic fermentation were 2, 3—butanediol, glycerol, valine and succinic acid (see in S2 File.).

In the wine of each cultivar the amount of ethanol, although quantitatively the most abundant metabolite, was similar and ethanol was not a major discriminant compound,in afct in my study,there is no enanol through procrsses. 2, 3—butanediol and glycerol are major constituents of wine. In this study the content of 2, 3—butanediol in Syrah wine was higher than in other wines and that compound appeared to be an important discriminant factor. The concentration of 2, 3—butanediol in the five wines in order from high to low was: Syrah > Cabernet Sauvignon > Zinfandel > Merlot > Ruby Cabernet. Glycerol was also an important discriminant compound. Its concentration in the five wines in order from high to low was: Zinfandel > Syrah > Cabernet Sauvignon > Merlot > Ruby Cabernet.

Most succinic acid in wine is a product of nitrogen metabolism by yeast but it is also formed during malolactic fermentation (MLF) in which lactic acid bacteria convert malate and citrate into lactate and other components [1]. Levels of succinic acid are thus related to the malic acid content of the grapes. The high level of lactic acid in each of the wines indicates MLF. The concentration of succinic acid is also generally related to the concentration of ethanol, glycerol and 2, 3—butanediol [9]. In a survey of the succinic acid content of Australian red wines made from Cabernet Sauvignon, Merlot, Ruby Cabernet and Shiraz between 1991 and 2003, Coulter et al. [35] found no influence of cultivar. In our study the range in concentration of succinic acid in wines was low and the order from high to low: Zinfandel > Ruby Cabernet > Cabernet Sauvignon > Merlot > Syrah. Succinic acid is very stable and its concentration changes little during aging [1] thus its close association with malic acid, a cultivar-related trait [36] indicates that it is likely to be a useful discriminant of wines according to cultivar. Interestingly Kliewer et al. reported that the level of malic acid in Zinfandel was about 70% greater than that in Merlot and Cabernet Sauvignon. That may explain the relatively high levels of succinic acid in Zinfandel wines in our study.

Having demonstrated the impact of plant genotype alone in discriminant analysis of ^1^H NMR spectra at one geographic site, we note that due to the biological interaction of genotype with environment both the discriminant factors and the degrees of discrimination obtained using the ^1^H NMR-PCA/PLS-DA approach are subject to specific environmental (edaphic, climatic, biotic and phyto-cultural) conditions at other sites as previous cross-environment studies have shown. It is this biological interaction that makes ^1^H NMR-PCA/PLS-DA a particularly powerful tool in the geographic discrimination of wine and other plant-based products.

Finally, our study indicates that ^1^H NMR-derived spectra of wine of single grape cultivars grown under the same conditions may be used in tandem with hierarchical cluster analysis to elucidate presently unknown genetic lineages and metabolomic relations of grapevine cultivars. In the absence of genetic information, for example, where predecessor varieties are no longer extant, this may be a particularly useful approach. Further confirmation of this prospect will require validation with more fully genetically-characterized cultivars.

## Conclusions

It may be concluded from this study that differences in grape-derived metabolites associated with genetic differences alone are a major source of 1H NMR-based discrimination of wines and that ^1^H NMR has the capacity to discriminate between very closely related cultivars.

The study also indicates that ^1^H NMR-derived spectra of wine of single grape cultivars grown under the same conditions coupled with hierarchical cluster analysis may be used to elucidate genetic lineages and metabolomic relations of grapevine cultivars.

## Supporting Information

S1 File Metabolites for PDO Lambrusco wine of Modena (Lambrusco Salamino di Santa Croce) DOI: 10.1021/jf302728b. (PDF)

S2 File Figures of compounds, PCA and PLS-DA to show discrimination between cultivars. DOI: 10.1016/j.foodres/2009.08.006.
(PDF)
