# Supplementary material for: Proton Nuclear Magnetic Resonance-Spectroscopic Discrimination of Wines Reflects Genetic Homology of Several Different Grape (V. vinifera L.) Cultivars
Source: PLoS One. 2015 Dec 11;10(12):e0142840. doi: 10.1371/journal.pone.0142840 (PMC4684234; doi:10.1371/journal.pone.0142840)

Appendix S1. Metabolites for PDO Lambrusco wine of Modena (Lambrusco Salamino di Santa Croce)

S1

$^{1}H-^{13}C$ heteronuclear multiple-bond correlation analyses.spectrumfor the wine

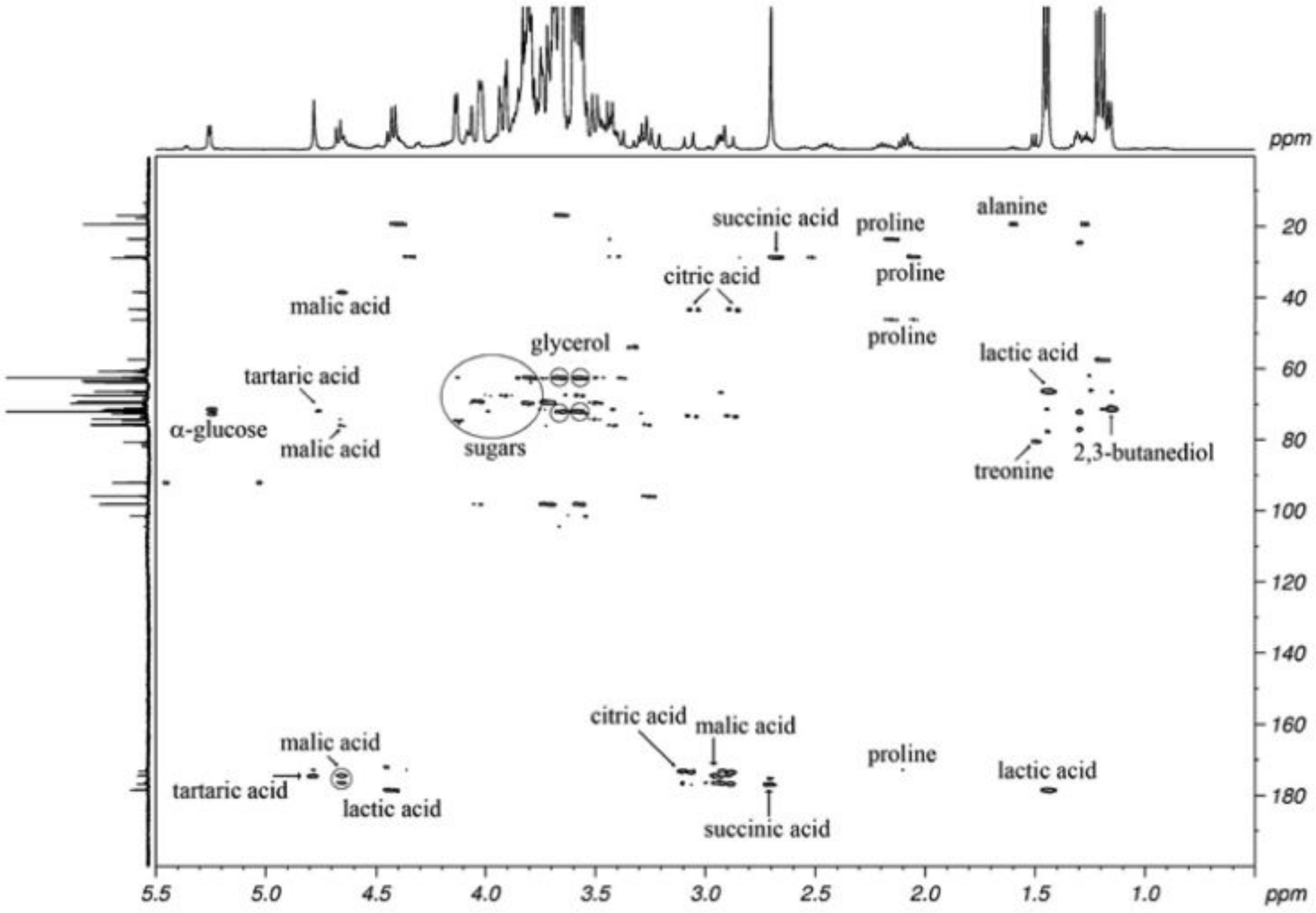

Supplement: S1 File — (PDF) [file pone.0142840.s001.pdf]
