# Supplementary material for: Proton Nuclear Magnetic Resonance-Spectroscopic Discrimination of Wines Reflects Genetic Homology of Several Different Grape (V. vinifera L.) Cultivars
Source: PLoS One. 2015 Dec 11;10(12):e0142840. doi: 10.1371/journal.pone.0142840 (PMC4684234; doi:10.1371/journal.pone.0142840)

Appendix S2.Figures of compounds, PCA and PLS-DA to show discrimination between cultivars

S2

$^{1}$H NMR spectra of wines vinified with Muscat Bailey A (A), Campbell Early (B), Kyoho (C), and Meoru (D) grapes grown in Korea.

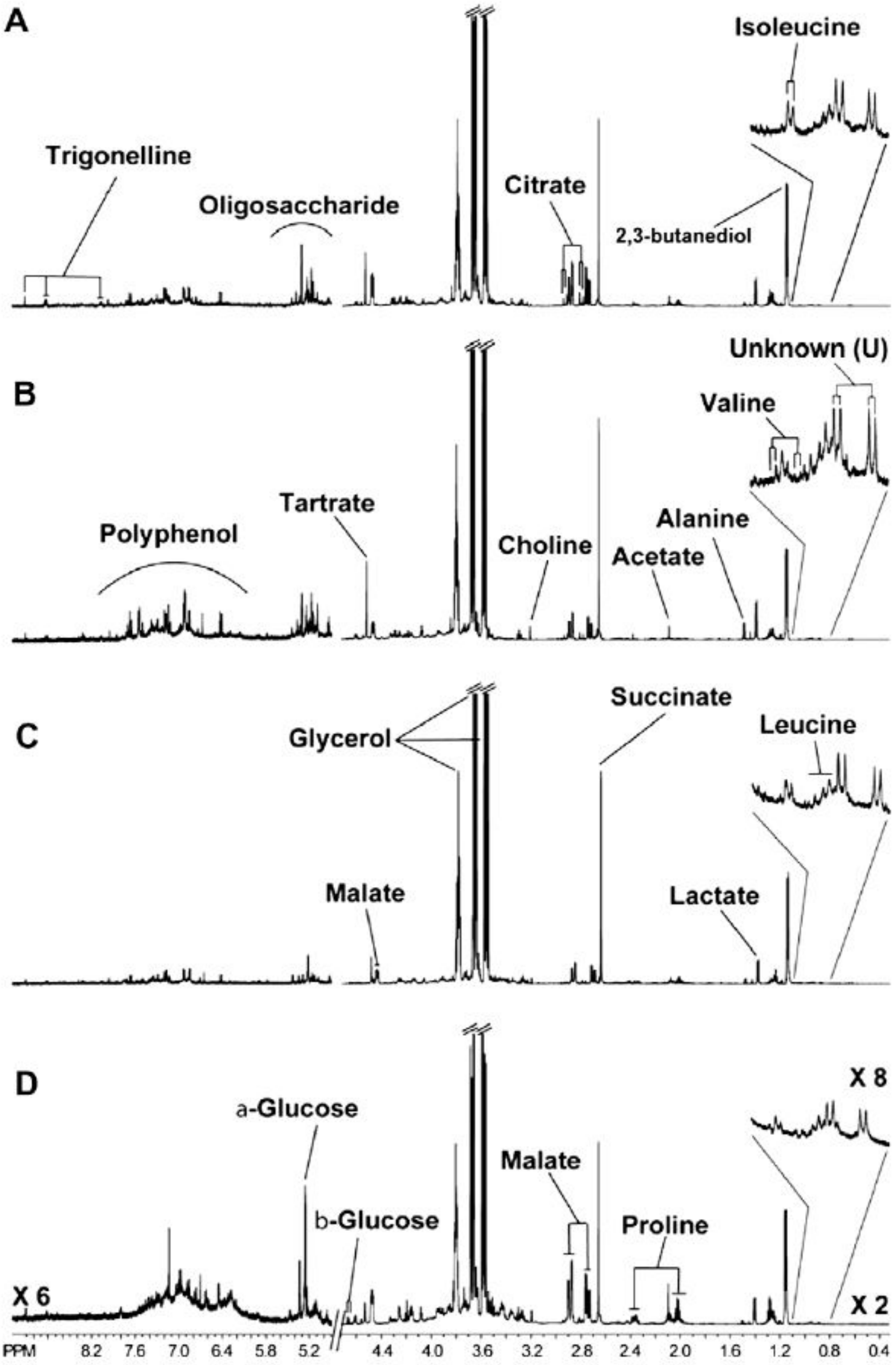

S2

PCA score plot for these 4 cultivars;

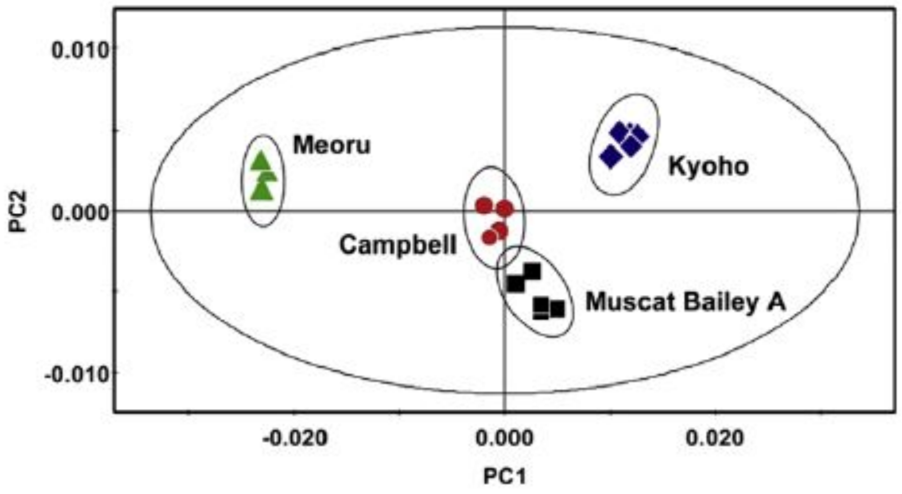

S2

Separations between wines vinified from Muscat Bailey A grapes and Campbell Early grapes (A), Muscat Bailey A and Kyoho grapes (B), and Campbell Early and Kyoho grapes (C)

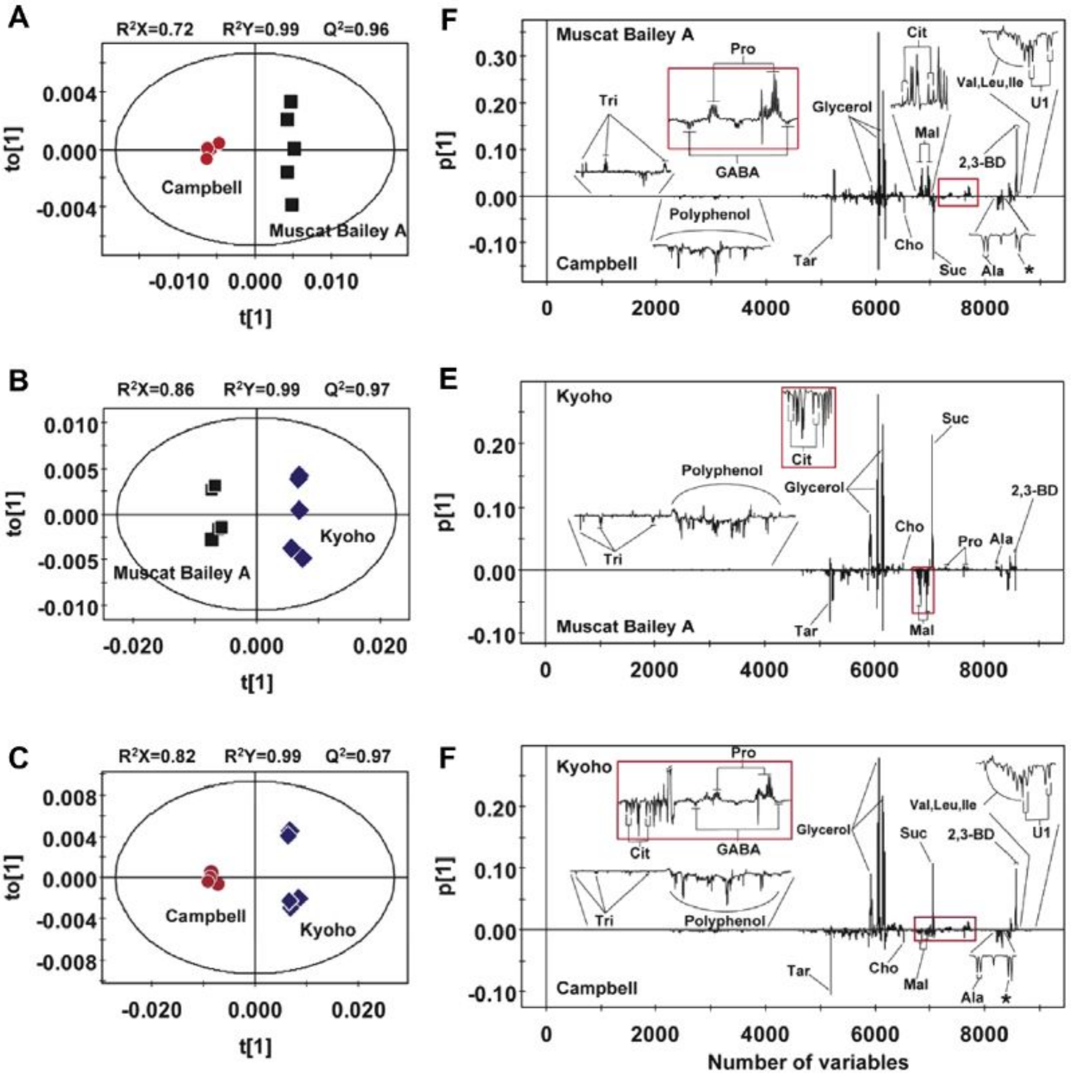

Supplement: S2 File — DOI: 10.1016/j.foodres/2009.08.006. (PDF) [file pone.0142840.s002.pdf]
